# *S*-maltoheptaose targets syndecan-bound effectors to reduce smoking-related neutrophilic inflammation

**DOI:** 10.1038/srep12945

**Published:** 2015-08-10

**Authors:** David CL Lam, Stanley CH Chan, Judith CW Mak, Craig Freeman, Mary SM Ip, Daisy KY Shum

**Affiliations:** 1 Department of Medicine, LKS Faculty of Medicine, The University of Hong Kong, HKSAR, China.; 2 Department of Biochemistry, LKS Faculty of Medicine, The University of Hong Kong, HKSAR, China; 3 Department of Pharmacology & Pharmacy, LKS Faculty of Medicine, The University of Hong Kong, HKSAR, China; 4 Department of Immunology, John Curtin School of Medical Research, Australian National University, Canberra, Australia

## Abstract

Cigarette smoke induces injury and neutrophilic inflammation in the airways of smokers. The stability and activity of inflammatory effectors, IL8 and neutrophil elastase (NE), can be prolonged by binding to airway heparan sulfate (HS)/syndecan-1, posing risk for developing chronic obstructive pulmonary disease(COPD). We hypothesize that antagonizing HS/syndecan-1 binding of the inflammatory effectors could reduce smoking-related neutrophil-mediated airway inflammation. Analysis of bronchoalveolar lavage fluid(BALF) of COPD patients found both total and unopposed NE levels to be significantly higher among smokers with COPD than non-COPD subjects. Similar NE burden was observed in smoke-exposed rats compared to sham air controls. We chose sulfated-maltoheptaose(SM), a heparin-mimetic, to antagonize HS/sydecan-1 binding of the inflammatory mediators in airway fluids and lung tissues of the smoke-exposed rat model. Airway treatment with SM resulted in displacement of CINC-1 and NE from complexation with bronchio-epithelial HS/syndecan-1, dissipating the chemokine gradient for neutrophil flux across to the bronchial lumen. Following SM displacement of NE from shed HS/syndecan-1 in bronchial fluids, NE became accessible to inhibition by α_1_-antitrypsin endogenous in test samples. The antagonistic actions of SM against syndecan-1 binding of NE and CINC-1 in smoke-exposed airways suggest new therapeutic opportunities for modulating airway inflammation in smokers with SM delivery.

Chronic obstructive pulmonary disease (COPD) results from smoke-induced injury in the airways and the inflammatory response may become self-perpetuating even in the absence of ongoing stimuli^1^. A key component in the pathophysiological process of airway inflammation in COPD is neutrophilic inflammation^2^. The vicious cycle of neutrophil influx and activation, release of neutrophil elastase (NE), and induction of further release of the chemokine interleukin 8 (IL-8) from activated bronchial epithelial cells and neutrophils reinforces an amplified, inflammatory cascade mechanism in smokers^3^,^4^. The binding of IL-8^5^ and NE^6^,^7^ by heparan sulfate (HS)/syndecan-1 would prolong stability and sustain activities of these inflammatory mediator proteins. Targeting HS/syndecan-1 binding of these effectors in the airway environment could modulate smoking-related airway inflammation.

Immobilisation and dimerisation of IL-8 on HS/syndecan-1^5^ that lined the endothelium and bronchial epithelium^8^ facilitated a chemokine gradient for neutrophil egress into the airway^9^. Elevated IL-8 levels in sputum and bronchial epithelium of patients with COPD correlated with the extent of neutrophilic inflammation and disease severity^10^,^11^,^12^.

While shedding of syndecan-1 dissipated the chemokine gradient^13^, binding of shed syndecan-1 to NE upon neutrophil degranulation^6^,^14^ sustained the activity of NE. Although local production of secretory leucoprotease inhibitor^15^ can counteract NE activity, the local effect can be overwhelmed at bronchial sites where neutrophilic inflammation recurs. Alpha_1_-antitrypsin (α_1_-AT), derived from not only hepatocytes^16^ but also from lung epithelial cells and alveolar macrophages^17^ would then become the major anti-protease defense^7^. Despite adequate production of anti-NE in COPD, NE could still evade anti-NE action by association with de-condensed chromatin^18^ and shed syndecan^6^.

Heparin fragments of <10-mer can compete with endogenous HS/syndecan-1 for binding to IL-8^5^ and NE^6^. Clinical application of such anti-inflammatory actions, however, was limited by the potent anti-coagulant property with potential to cause haemorrhage^19^. That 2-O, 3-O-desulfated heparin showed minimal anti-coagulant activity but anti-protease and anti-inflammatory activities in a murine model of NE-induced airway inflammation^20^ added impetus to the pursuit of oligosaccharide alternatives.

In this study, we assessed BALF samples from smokers/ex-smokers with and without COPD for unopposed versus total NE burden and IL-8 and confirmed that findings were similarly observable in a rat model of smoking-induced lung inflammation. Sulfated-maltoheptaose (*S*-maltoheptaose) was tested as a heparin mimetic to target associations of airway NE and CINC-1 (the closest functional counterpart of human IL-8 in rats)^21^ with HS/syndecan-1. In smoke-exposed rats, airway delivery of *S*-maltoheptaose antagonized HS/syndecan-1 binding of NE and CINC-1 thus providing for dampened activities of the effectors in neutrophilic inflammation of the airways.

## Results

### Unopposed NE activity in BALF of COPD patients

**Figure 1:**
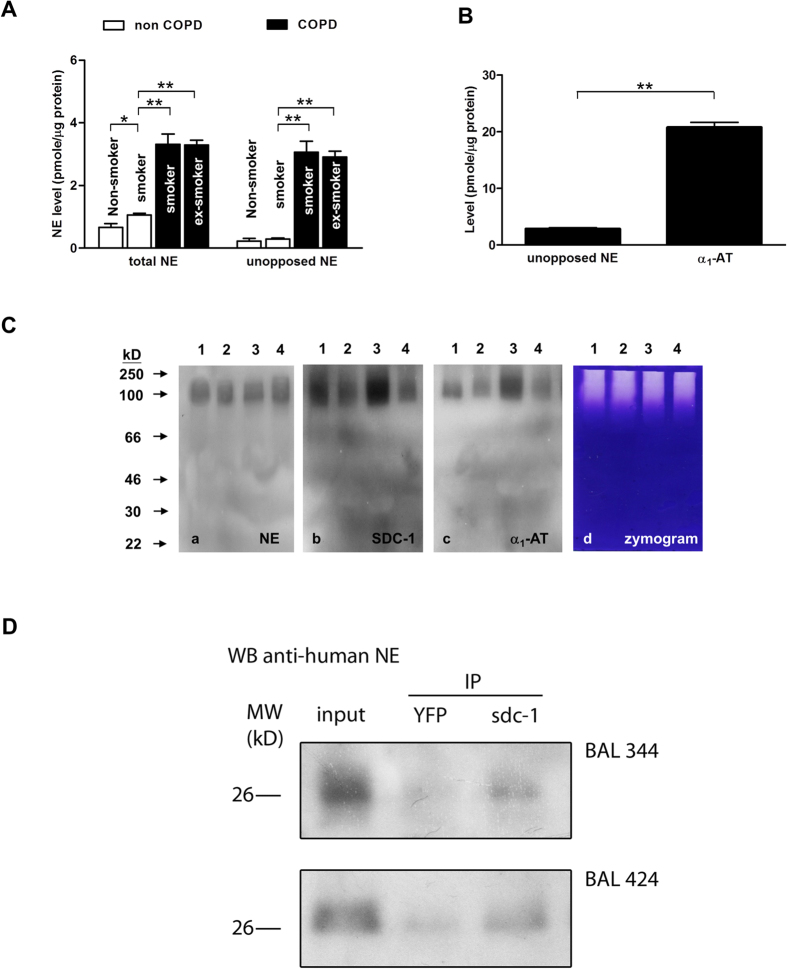
NE is unopposed in BALF of patients with COPD. (**A**) BALF samples were assayed for NE levels, both total (by ELISA) and unopposed (by active site titration). COPD patients (current smokers, n = 20; ex-smokers, n = 8) indicated higher total and unopposed NE levels than the non-COPD controls (n = 14). In COPD, given the near equivalence of total NE and unopposed NE, NE in bronchial environment existed in forms unopposed by physiological anti-elastase. (**B**) These forms were unopposed despite eight times of the endogenous elastase inhibitor, α_1_-AT. **P < 0.001, *P < 0.01. (**C**) Western blots of BALF of representative COPD patients (lanes 1 – 4) probed by antibodies against NE (a), syndecan-1 (SDC-1), (b) and α_1_-AT (c); corresponding casein zymogram of the BALF samples (d). (**D**) Specific antibody against syndecan for immunoprecipitation of the syndecan-NE complex from BALF samples of representative COPD patients, followed by SDS-PAGE in attempt to dissociate syndecan from NE and then immunoblotting assay with the same NE antibody again. Western blotting demonstrated unbound NE (dissociated from the syndecan-NE complex) at the expected band size of 29 kDa. Although the band width appeared to be a smear, the peak density corresponded to the expected band size at 29 kDa. Representative results from two BALF samples of COPD patients are shown.

The BALF of 42 subjects (28 with COPD and 14 non-COPD controls) were eligible for analysis; the difference in mean age between the two groups (COPD and non-COPD) were not statistically significant (P = 0.065) (Table 1). The levels of unopposed and total NE in BALF from COPD patients were significantly higher than those in subjects without COPD, irrelevant of smoking status (Fig. 1A). COPD patients had significantly higher NE levels compared to non-COPD subjects, and unopposed NE approximated that of total NE (Fig. 1A) despite the molar excesses of α_1_-AT to unopposed NE at ratios averaging 8:1 (Fig. 1B). Among the non-COPD subjects, differences in total NE levels between the non-smokers and current smokers were statistically significant (P < 0.01) but differences in unopposed NE between these two groups of subjects were not (P = 0.137) (Fig. 1A). Western blots of BALF from COPD patients indicated co-localisation of NE, syndecan-1 and α_1_-AT in the complex (nominal range of 90–250 kDa) while the corresponding casein zymogram reinforced occurrence of unopposed NE activity even though α_1_-AT was associated with this complex (Fig. 1C). To confirm NE-syndecan interaction in the patient samples, we used a specific antibody against syndecan-1 for immunoprecipitation of the syndecan-NE complex from BAL samples of COPD patients. Analysis of the recovered immunoprecipitate by SDS-PAGE under reducing condition achieved dissociation of syndecan-1 from NE as revealed in the Western blot where NE-immunoreactivity was observable at the expected molecular size (29 kD) (Fig. 1D). The procedure was repeated with use of an irrelevant antibody, that against YFP, as negative control. Only a faint band was observable at the position corresponding to free NE (Fig. 1D). We therefore confirmed the occurrence of unopposed NE activity in syndecan-NE complexes found in BAL samples of COPD patients.

### Unopposed NE activity in BALF of the smoke-exposed rat model

**Figure 2:**
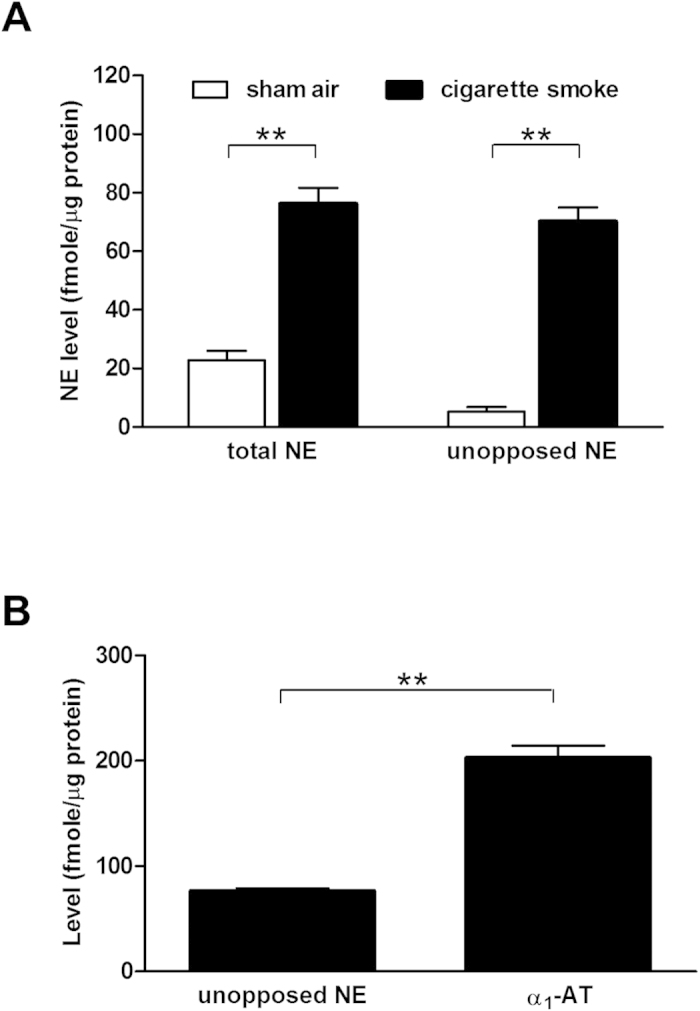
NE is unopposed in BALF of cigarette smoke-exposed rats. (**A**) Cigarette smoke-exposed rats (n = 8) indicated higher NE levels, both total and unopposed, than those of the sham air controls (n = 8). (**B**) These forms were unopposed despite three times of the endogenous inhibitor, α_1_-AT. **P < 0.001.

Following exposure to cigarette smoke, unopposed NE rose to as high a level as total NE in the BALF whereas the sham air controls indicated a basal level of unopposed NE that was significantly lower than the total NE (Fig. 2A). Smoking-induced unopposed NE was not due to inadequate supply of α_1_-AT as shown by the excess of α_1_-AT to unopposed NE at molar ratios averaging 3:1 in the BALF of smoke-exposed rats (Fig. 2B).

### Decrease in unopposed NE activity with *S*-maltoheptaose treatment

**Figure 3:**
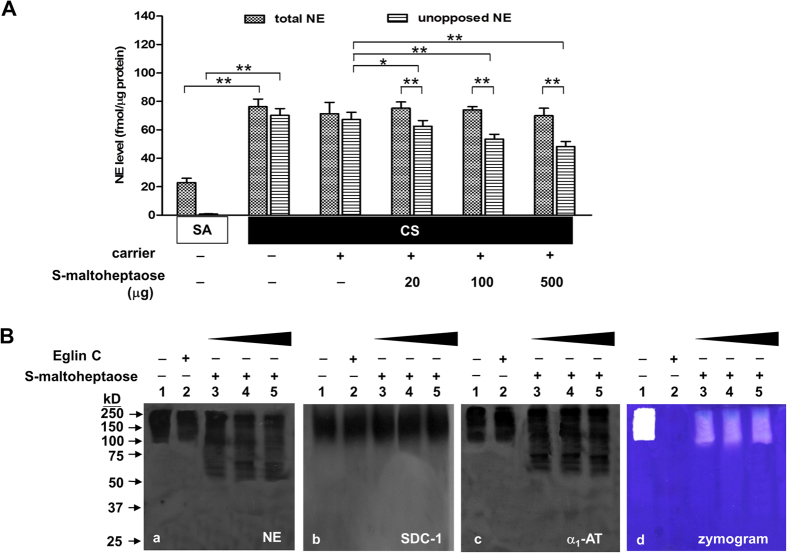
Dose-dependent decrease in unopposed NE with airway treatment of *S*-maltoheptaose. (**A**) Airway treatment of cigarette smoke-exposed rats (CS, n = 8) with *S*-maltoheptaose-on-chitosan carrier beads resulted in dose-dependent decrease in unopposed NE despite negligible change in total NE in BALF samples. NE levels of the sham air controls (SA, n = 8) are included for comparison. **P < 0.001, *P < 0.01. (**B**) Western blots of the BALF samples were performed for NE (a), syndecan-1 (SDC-1), (b) and α_1_-AT (c); casein zymography of the BALF samples was performed in parallel (d). NE and α_1_-AT remained co-localized with SDC-1 in the nominal range of 90–250 kD (Lanes 1 and 2 of each panel); this range indicated proteolytic activity (panel d, Lane 1) that was inhibited by treatment with Eglin C tetrapeptide (Panel d, Lane 2). In BALF samples of rats given airway treatment of *S*-maltoheptaose, NE- and α_1_-AT-immunopositivites extended beyond the SDC-positive region (Panels a–c, Lanes 3–5) in correlation with no proteolytic activity in the casein zymogram (Panel d, Lanes 3–5). When NE was displaced from SDC-1, it became accessible to inhibition by α_1_-AT. Lanes 1: neat BALF; Lanes 2: Eglin c-treated BALF; Lanes 3–5: BALF from smoke-exposed rats treated respectively with 20 μg, 100 μg and 500 μg of *S*-maltoheptaose on carrier beads via the airways.

Following delivery of *S*-maltoheptaose to the airways of smoke-exposed rats, dose-dependent decrease in unopposed NE reached as low as 65% of the levels in smoke-exposed rats which were given blank carrier without *S*-maltoheptaose (Fig. 3A). Total NE, however, remained at elevated levels, similar to those of smoke-exposed rats treated with blank carrier only (without *S*-maltoheptaose added). In the sham air group, both total and unopposed NE remained at basal levels with *S*-maltoheptaose treatment.

Western blot and zymographic analyses of BALF samples were performed to explore if *S*-maltoheptaose acted by displacement of NE from association with syndecan-containing complexes (Fig. 3B). In BALF of smoke-exposed rats treated with blank carrier only (without *S*-maltoheptaose), NE in co-localization with α_1_-AT and syndecan-1 in the nominal range of 90–250 kD in the Western blot (Fig. 3B, Lanes 1 in panels a – c) was found to be proteolytically active in the casein zymogram (Fig. 3B, Lane 1 in panel d). In the presence of NE-specific Eglin C tetrapeptide, the proteolytic activity was no longer detectable (Fig. 3B, Lane 2 in panel d) whereas associations of NE and α_1_-AT with syndecan-1 persisted (Fig. 3B, Lanes 2 in panels a – c). In BALF of smoke-exposed rats treated with increasing dose of *S*-maltoheptaose, NE- and α_1_-AT-immunopositivities extended into the (50–90 kD) region in the Western blot, beyond the syndecan-positive region (Fig. 3B, Lanes 3–5 in panels a and c). The corresponding (50–90 kD) regions of the casein zymogram indicated no proteolytic activity in contrast to residual proteolytic activity observable in the (90–250 kD) regions (Fig. 3B, Lanes 3 – 5 in panel d).

### Decreased neutrophil count and myeloperoxidase with *S*-maltoheptaose treatment

**Figure 4:**
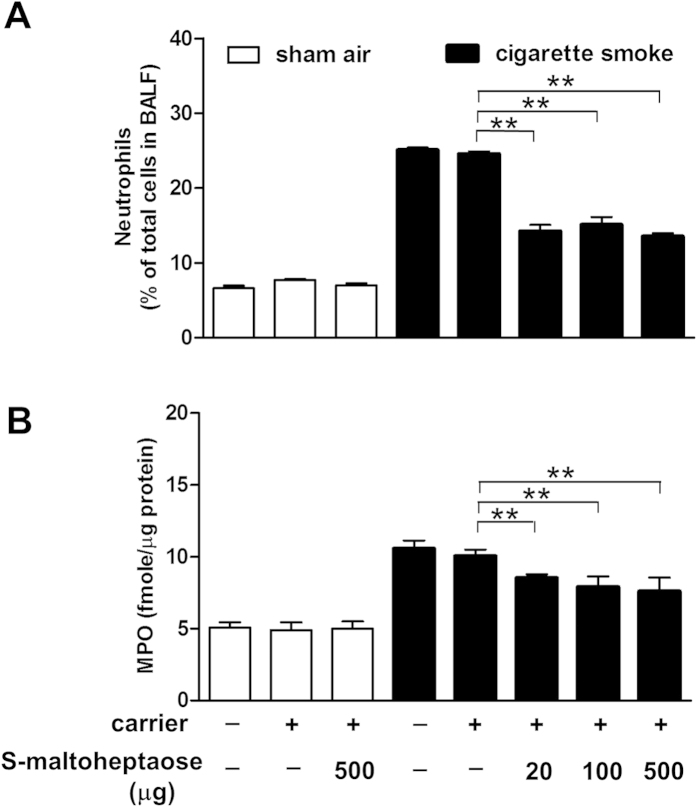
Neutrophil recruitment and degranulation into the bronchial lumen level off with airway treatment of *S*-maltoheptaose. Cigarette smoke-exposed rats (CS, n = 8) were given airway treatment of *S*-maltoheptaose-on-chitosan carrier beads at the doses indicated. The CS group showed significant increase in neutrophil count (**A**) and myeloperoxidase (MPO, neutrophil degranulation marker) (**B**) when compared with basal values of sham air controls (SA, n = 8). In both groups, the increase in basal levels of the SA group were unaffected by airway treatment with the carrier beads with or without loading of *S*-maltoheptaose. **P < 0.001.

Following delivery of *S*-maltoheptaose to the airways of smoke-exposed rats, the rise in percentage of neutrophils in BALF over the sham air group (25.08 ± 0.77% vs 6.60 ± 0.84% respectively) was halved in the dose range tested. By contrast, percentage of neutrophils remained high in the smoke-exposed rats that were treated with blank carrier only (Table 2 and Fig. 4A). Neither treatment with *S*-maltoheptaose nor the blank carrier affected the inflammatory cell counts or percentage of neutrophils in the sham air group (Table 2 and Fig. 4A). Within the dose range of *S*-maltoheptaose tested, MPO activity in the BALF of smoke-exposed rats dropped by 20%, contrasting the elevated MPO activities among smoke-exposed rats treated with blank carrier or otherwise (Fig. 4B).

### Immunohistochemistry for neutrophils and CINC-1 in rat bronchial tissue

**Figure 5:**
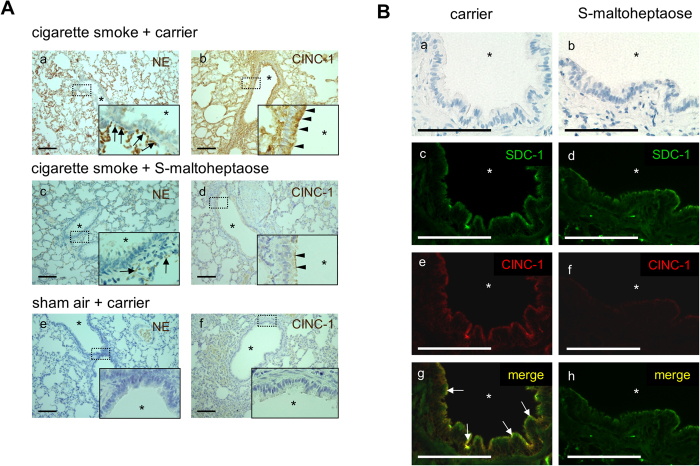
Decrease in airway accumulation of neutrophils and CINC-1 following treatment with *S*-maltoheptaose. (**A**) Lung tissue sections were stained for NE (a, c, e) or CINC-1 (b, d, f) and then counter-stained with haematoxylin. NE-positive cells lining the basal side of the bronchial epithelium were rarely observed in cases treated with *S*-maltoheptaose (a vs c, arrows). CINC-1 immuno-positivity enriched along the luminal and basal sides of the bronchial epithelium were barely observable in cases treated with *S*-maltoheptaose (b vs d, arrowheads). Neither immuno-positivity for NE nor CINC-1 was observable in the sham air controls (e & f). (**B**) Double immunofluorescence for syndecan-1 (SDC-1) and CINC-1. (a) and (b): Bright field images of the lung tissue sections. Tissues treated with mere carrier revealed localisation of SDC-1 (c) and CINC-1 (e) to the bronchial epithelium, more prominent on the luminal side than on the basal side. The merged image (g) further revealed co-localisation of SDC-1 and CINC-1. Tissue sections treated with *S*-maltoheptaose indicated weaker immunofluorescence for SDC-1 (d) and hardly any detectable CINC-1 (f) at the bronchial epithelium. This was reinforced by the sole immunofluorescence of SDC-1 at the bronchial epithelium in the merged image (h). Arrows in (g) indicate sites where SDC-1 and CINC-1 are co-localised at the luminal side of the bronchial epithelium. *bronchial lumen. Scale bar = 100 μm.

In contrast to the dense accumulation of neutrophils along the vascular endothelium adjacent to the basal side of the bronchial epithelium as shown by smoke-exposed rats treated with blank carrier (Fig. 5A, panel a), airway treatment with *S*-maltoheptaose resulted in marked reduction of neutrophils (Fig. 5A, panel c). The minimal neutrophils in airways of the sham air controls were unaffected by treatment with carrier, loaded with *S*-maltoheptaose or without (Fig. 5A, panel e).

In smoke-exposed rats treated with *S*-maltoheptaose, CINC-1 immuno-reactivity was weak on the luminal side and hardly detectable on the basal side (Fig. 5A, panel d). This contrasts with the dense CINC-1 immuno-reactivity along both luminal and basal sides of the bronchial epithelium as observable in smoke-exposed rats with blank carrier (Fig. 5A, panels b versus d). Double immunofluorescence further revealed co-localisation of CINC-1 and syndecan-1 along the bronchial epithelium in the positive controls (Fig. 5B, panels c, e, g). Treatment with *S*-maltoheptaose resulted in barely detectable CINC-1 immuno-reactivity whereas syndecan-1 immuno-reactivity remained detectable along the bronchial epithelium.

### S-maltoheptaose selectively targeted the CINC-1 gradient

**Figure 6:**
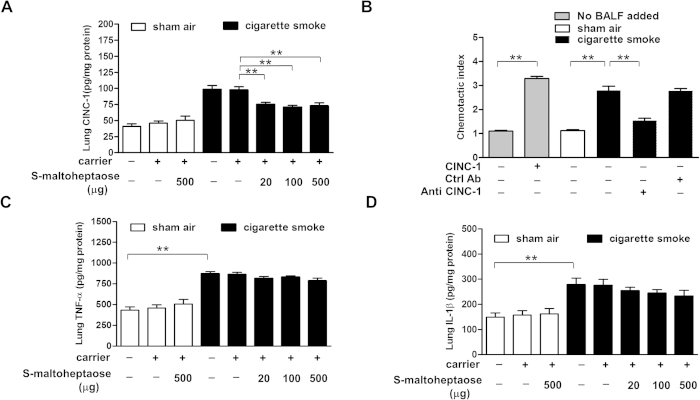
CINC-1 but neither TNF-α nor IL-1β in lung homogenates levels off following treatment with *S*-maltoheptaose. Cigarette smoke-exposed rats (CS, n = 8) and sham air controls (SA, n = 8) were given airway treatment of *S*-maltoheptaose-on-chitosan carrier beads at the doses indicated. The levels of CINC-1 (**A**), TNF-α (**C**) and IL-1β (**D**) in lung homogenates were determined with ELISA. The CS group showed significant increase in the three cytokines when compared with basal levels in lung homogenates of the SA group. The increase in CINC-1 was lowered to the 50^th^-percentile level with treatment of *S*-maltoheptaose but the increase in TNF-α and IL-1β were maintained. Basal levels of the SA group were unaffected by airway treatment with the carrier beads with or without loading of *S*-maltoheptaose. (**B**) BALF samples of both CS and SA groups were subjected to Boyden chamber assay for neutrophil chemotactic activity due to CINC-1. The chemotactic index of the CS group was significantly higher than that of the SA group. Pretreatment of the CS sample with an antibody against CINC-1 at 50 ng/ml maximally neutralized 72% of the neutrophil chemotactic index. **P < 0.001.

To verify that *S*-maltoheptaose perturbed the gradient of CINC-1 across the bronchial epithelium, lung tissue homogenates of smoke-exposed rats were monitored for change in CINC-1 level. The rise in CINC-1 level in the smoke-exposed rats above that of sham air controls (98.39 ± 17.25 pg/mg protein vs 41.02 ± 10.01 pg/mg protein, respectively) was reduced by 50% following treatment with 20 μg *S*-maltoheptaose (Fig. 6A). No further decline in CINC-1 level was seen with increase in *S*-maltoheptaose dose.

Among the smoke-exposed rats, the CINC-1 levels in BALF from sham-treated rats measured 196.50 ± 20.63 ng/ml, whereas those following *S*-maltoheptaose treatment measured 265.00 ± 19.34 ng/ml (n = 3 in each case, P < 0.01). Chemotactic response to CINC-1 in BALF samples of smoke-exposed and control rats were assessed with use of the Boyden chamber. The chemotactic index shown by neutrophils in response to recombinant CINC-1 in medium (10 ng/ml) was similar to that in response to smoke-exposed rat BALF. The chemotactic response to smoke-exposed rat BALF was suppressed maximally by 72% following neutralisation with a CINC-1-specific antibody at 50 ng/ml (Fig. 6B). In contrast, a non-specific antibody showed no neutralisation effect.

We also examined the smoke-exposed rat lung tissue homogenates for pro-inflammatory cytokines that do not bind HS moieties in the airways, such as TNF-α and IL-1β. The rise in TNF-α level in the smoke-exposed rats above that of the sham air group (871.74 ± 99.84 pg/mg protein vs 433.54 ± 93.15 pg/mg protein) was maintained following treatment with *S*-maltoheptaose within the dose range tested (Fig. 6C). Similarly, the rise in IL-1β level in smoke-exposed rats over that of the sham air group (278.27 ± 54.11 pg/mg protein vs 149.31 ± 46.98 pg/mg protein) was maintained following the same dose range of *S*-maltoheptaose treatment (Fig. 6D).

## Discussion

In the BALF of COPD subjects and of smoke-exposed rats, we demonstrated unopposed NE activity in the presence of an excess of α_1_-AT. In both cases, association of NE with HS/syndecan-1 precluded inhibition of NE activity by the prevailing α_1_-AT. The delivery of *S*-maltoheptaose to the airways of smoke-exposed rats could compete out NE and CINC-1 from associations with HS/syndecan-1.

The unopposed NE was high in BALF from smokers with COPD compared to non-smokers or smokers without COPD. The results reflected perpetuation of neutrophil extravasation and degranulation in the airway environment even after cessation of smoking in COPD. These results are consistent with previous findings in both longitudinal^22^ and cross-sectional studies^23^,^24^ that elevations in neutrophils and IL-8 in sputum persisted in ex-smokers with COPD. Such unopposed or excessive NE activities have also been reported in ex-smokers with subclinical emphysema^12^. Recurrent airway exposure to cigarette smoke induces pro-inflammatory cytokines such as IL-1β and TNF-α, which in turn induce a chemokine gradient^25^. We have previously demonstrated in bronchiectasis that syndecan-binding of NE in sputum sol was responsible for limiting access to NE by prevailing α_1_-AT, resulting in high unopposed NE activity^6^,^7^. We speculate that in COPD, the smoke-induced airway remodeling has progressed to support a chemokine gradient which sustains egress of neutrophils into the airways and allows NE activity that evades opposition by α_1_-AT. Here in this work we confirmed that in the BALF of COPD subjects, NE existed in syndecan-bound forms that fail to be opposed by α_1_-AT.

Our choice of *S*-maltoheptaose^26^ is based on preliminary finding of *in vitro* efficacy of the sulfated malto-oligosaccharide series in displacement of NE from the syndecan complex in BALF of COPD subjects, increasing up the series from maltose to maltoheptaose. In the rat model, airway treatment with *S*-maltoheptaose not only displaced NE from binding to syndecan-1 complexes in the bronchial lumen, but also facilitated encounters of displaced NE with prevailing α_1_-AT to effect inhibition of NE activity. The decrease in unopposed NE observed in BALF after *S*-maltoheptaose treatment was dose-dependent but the post-treatment levels did not reach basal levels, indicating the potential for dose adjustment or titration to levels that can prevent tissue destruction yet preserve innate immunity due to NE activity. These results suggest the need for further experiments with temporal spacing of doses, and evaluation of functional outcomes with lung function tests.

In the smoke-exposed rats, the rise in neutrophils in BALF was reduced despite increased doses of *S*-maltoheptaose, implying a low dose of *S*-maltoheptaose was sufficient for curbing neutrophil recruitment to the inflamed airways. Like NE, MPO is released from azurophilic granules of neutrophils upon neutrophil degranulation in COPD^27^. Dissimilar to NE, however, MPO activity was not confounded by binding to HS moieties of shed proteoglycans^28^. MPO activity in the BALF of smoke-exposed rats dropped by 20% with delivery of *S*-maltoheptaose, reflecting curbed recruitment of neutrophils to the airways at a low dose of *S*-maltoheptaose.

Airway treatment with *S*-maltoheptaose resulted in marked reduction of neutrophils along the basal side of the bronchial epithelium; together with the weak CINC-1 immuno-reactivity on the luminal side in smoke-exposed rat, these suggested the disruption of neutrophil extravasation into the airways with *S*-maltoheptaose treatment. These results supported that CINC-1 is a target molecule displaced from bronchio-epithelial syndecan-1 by *S*-maltoheptaose. As shown in the Boyden chamber experiments, the chemotactic response to smoke-exposed rat BALF was suppressed to a large extent by more than 70% following neutralisation with a CINC-1-specific antibody, supporting the role of rat CINC-1 as a major chemokine that recruited neutrophils to the bronchial epithelium of smoke-exposed rats. The maintained rise in TNF-α and IL-1β level in the smoke-exposed rats above that of the sham air group even after treatment with *S*-maltoheptaose within the dose range tested (Figs. 6C,D) supported that *S*-maltoheptaose targeted HS-binding effectors like NE and CINC-1 during neutrophilic inflammation in the bronchial environment.

Recent studies have demonstrated the importance of HS moieties of cell surface syndecan-1 in binding to chemokines and in establishing a chemotactic gradient which guides neutrophil extravasation to the inflamed airway^29^. Binding to HS moieties can protect IL-8 from proteolytic attack and thus prolong the half-life of the chemokine in the inflamed airways^30^. Airway delivery of IL-8 mutated in the HS binding domain led to decreased accumulation of the chemokine and neutrophils in lung tissue of otherwise healthy animals^31^,^32^. We exploited the concept that sulfated oligosaccharides of 6- to 10-mer are heparin mimetics that can compete with tissue HS moieties for binding of IL-8^33^ and thus can abolish the gradient of inflammatory chemokines. Taking advantage of the defined molecular size and negative charge of *S*-maltoheptaose for ready loading onto carrier chitosan beads by electrostatic attraction and its predictable affinities for HS-binding inflammatory effectors in airway environments, we showed that airway treatment of smoke-exposed rats with a low dose of *S*-maltoheptaose was sufficient to decrease neutrophil count and MPO level in BALF. The same low dose of *S*-maltoheptaose was sufficient to decrease CINC-1 in smoke-exposed rat lung tissue homogenate. Immunohistochemical staining indicated de-localisation of CINC-1 from the syndecan-1-positive lining of bronchial epithelium. Our results reinforce not only the importance of HS moieties in the inflamed airway tissue in maintaining the neutrophil chemotactic gradient but also suggest a strategy for dissipation of the aberrant chemokine buildup at HS moieties of the affected airway environment.

Other than *S*-maltoheptaose, synthetic heparin oligosaccharides can be synthesized with limited anti-factor Xa and anti-factor IIa activities^20^,^34^,^35^ and these could as well be exploited to target HS-binding effectors of neutrophilic inflammation. Sulfated linked cyclitols^26^ that are defined in molecular size and charge have also been designed to inhibit protein/HS interactions. Given a listing of these synthetic heparin mimetics according to affinities for HS-binding inflammatory effectors and preclinical indications of efficacy, informed selection of heparin mimetic(s) for treatment of aberrant neutrophilia and unopposed NE in the COPD airway environment will be possible^20^.

It is noteworthy that the short duration and passive tobacco smoke exposure in the rat model may not be the same as chronic tobacco smoking in human smokers. However, we did observe similar patterns of unopposed NE burden in relation to prevailing anti-protease in the BALF of COPD and the rat model.

In conclusion, we demonstrated that recurrent cigarette smoke exposure induced neutrophil accumulation and unopposed NE activity despite excess anti-protease activity in the rat airways, similar to that found in the airways of current and ex-smokers with COPD. *S*-maltoheptaose instilled into the airways of this rat model could compete out IL-8 and NE from respective associations with immobilised and shed forms of HS/syndecan-1, reducing neutrophil chemotaxis across the bronchial epithelium and rendering NE susceptible to inhibition by anti-protease. The animal data provides proof-of-concept for a possible strategy for treatment of chronic airway diseases in which NE activity is persistently dominant despite adequate anti-protease activity.

## Materials and Methods

### Subjects

The study protocol was approved by the Institutional Review Board of the Hong Kong University/Hong Kong Hospital Authority Hong Kong West Cluster (IRB UW 11-085). All clinical procedures were performed in accordance with approved regulations and guidelines. Successive subjects undergoing bronchoscopy in the Department of Medicine, Queen Mary Hospital, Hong Kong, were recruited. Recruited subjects were recorded, as current smokers, ex-smokers, or non-smokers. Written informed consent for collection of a sample of BALF for research was obtained prior to diagnostic bronchoscopy. Spirometry was performed according to the ATS/ERS criteria. The severity of airflow obstruction was classified according to GOLD Statement (www.goldcopd.com). Patients in acute exacerbation of COPD were excluded from bronchoscopy. BALF samples collected from subjects with endobronchial tumor or lesions, active pneumonia, asthma and bronchiectasis were excluded. BALF collections from smokers, ex-smokers and non-smokers who did not have evidence of airflow obstruction on spirometry served as controls.

### Lung function tests and collection of BALF from COPD subjects

The lung function tests included spirometric tests of forced vital capacity (FVC) and forced expiratory volume in one second (FEV_1_). Spirometry measurements were done using a Sensor-Medics V22d Pulmonary Function Lab System (SensorMedics, Yorba Linda, CA, USA). Bronchoalveolar lavage fluid (BALF) was collected from COPD subjects in a sub-segmental bronchus during bronchoscopy by instillation and aspiration of 150 ml sterile and pyrogen free phosphate buffered saline. To minimise cell contamination from the upper airways, the recovered BALF fluid was centrifuged at 200 g for 10 min at 4 °C. The supernatant was concentrated ten times with the use of Amicon Ultra-15 centrifugal filter Unit (Millipore, Billerica, MA, USA) at 400 g for 15 min. The concentrate was then divided into aliquots for storage at −80 °C until use.

### Preparation of chitosan beads and loading with sulfated maltoheptaose

*S*-maltoheptaose was prepared as described before^36^ and loading of chitosan beads with *S*-maltoheptaose was prepared as follows: Five-micron (5 μm) chitosan beads were prepared with use of SPG membrane emulsification^37^. Briefly, chitosan (1.6%) was prepared in a solution containing 1% (w/v) acetic acid and 5% (w/v) sodium chloride. The chitosan solution (2 ml) (aqueous phase) was forced (at 16 kPa) through 5-μm pores of the SPG membrane into the oil phase containing 4% (w/v) Tween® 80, with stirring of the W/O emulsion at 600 rpm for 2 hours. Chitosan beads that formed in the W/O emulsion were solidified by addition of a mixture made up of 100 μl of 10 M NaOH, 100 μl of Tween® 80 and 4 ml of mineral oil. Chitosan beads were washed with 1% Tween 80 (x2) and then distilled water (x3). After air-drying, the chitosan beads were stored under dessication at 24 °C.

In preparation for use, the chitosan beads (50 mg) were re-suspended in 1 ml of PBS (pH 5.8) containing 1 mg/ml of sulfated maltoheptaose at 24 °C for 16 hours. The mixture was centrifuged (15,000 g, 2 min) and then 0.1 ml of the supernatant was sampled to assay for the sulfated maltoheptaose that remained in the supernatant with use of CPC turbidimetry^38^. Sulfated maltoheptaose that was loaded onto the chitosan beads was determined by the difference between the total sulfated maltoheptaose added to the suspension of chitosan beads and that which remained in the supernatant after the 16-hour incubation.

### Airway delivery of *S*-maltoheptaose to smoke-exposed rats

Sprague-Dawley rats (200 g body weight) were divided into groups of twelve: (i) smoke-exposed rats (exposed to 4% cigarette smoke: Camel brand; filter, R.J. Reynolds, Winston-Salem, NC, USA), one hour/day for 30 days, in a ventilated smoking-chamber^39^; (ii) sham air control rats inhaled room air. Under pentobarbitone anesthesia (60 mg/kg body weight; intraperitoneal), six rats in each group were given intra-tracheal administration of *S*-maltoheptaose (20 – 500 μg)-loaded chitosan beads (2 mg) via an insufflator device (PennCentury Inc., Philadelphia, Pennsylvania, USA). The other six rats in each group received neat chitosan beads (2 mg) as vehicle control. After recovery from anaesthesia, rats accessed food and water *ad lib* until sacrifice 72 hours later under pentobarbitone overdose. BALF and lung tissues were collected for analysis. All procedures were in strict accordance with the NIH Guide for the care and use of laboratory animal and approved by the Committee on Use of Live Animals for Teaching and Research, LKS Faculty of Medicine, The University of Hong Kong.

### Assay for unopposed NE concentration in BALF

The unopposed NE activities, expressed as molar ratios both to total NE and to α1-antitrypsin as determined by ELISA, were assayed in BALF of COPD patients and non-COPD controls, and in BALF of smoke-exposed rats compared to sham air controls, before and after airway administration of *S*-maltoheptaose. The unopposed NE activities was determined spectrophotometically by using MeO-Suc-Ala-Ala-Pro-Val-p-nitroanilide as substrate at 2 mM in 0.2 M Tris-HCl, 0.5 M NaCl, pH 8.0; hydrolytic release of p-nitroaniline was followed spectrophotometrically at 410 nm, 37 °C (E410 = 8800). To determine the equivalent molarity of active NE, the rate of p‐nitrophenol production from the samples was monitored and reference to that of equivalent activity of purified NE (Merck Millipore, Germany), of which the active concentration have been titrated with increasing known concentrations of the NE irreversible inhibitor, methoxysuccinyl-l-alanyll-alanyl-prolyl-l-valyl-chloromethylketone. The minimum concentration of the inhibitor that can completely inhibit the activities gave the active site concentration of the purified NE^7^.

### Assay for total NE concentration in BALF

NE was measured with a sandwich enzyme-linked immunosorbent assay (ELISA) kit (Hycult Biotech, The Netherlands) according to the manufacturer’s instructions.

### Assay for MPO Activity in BALF

Myeloperoxidase (MPO) activity of BALF was assayed spectrophotometrically using 0.0005% hydrogen peroxide as substrate in 50 mmol/L phosphate buffer containing 0.167 mg/ml o-dianisidine dihydrochloride at pH 7.0. The change in absorbance at 460 nm, 37 °C was measured using a spectrophotometer. The MPO activity was then determined by reference to commercial available MPO (Merck KGaA, Darmstadt, Germany) which had been standardized by active site titration. One unit of MPO activity was defined as that degrading 1 μmole of peroxide per minute at 37 °C. Data were expressed as mole per unit gram of protein.

### Assay for α1-AT concentration in BALF

Sandwich enzyme-linked immunosorbent assay (ELISA) for α_1_-AT level in BALF was performed with goat anti-human α_1_-AT (Sigma-Aldrich, St. Louis, MO, USA) immobilised in the wells of microtitre plates. Non-specific binding was blocked with 3% bovine serum albumin. Known dilutions of BALF were applied at 100 μl/well and incubated (1 h, 37 °C) to allow α_1_-AT capture. The captured antigen was incubated (1 h, 37 °C) in turn with the primary antibody, rabbit anti-human α_1_-AT (diluted 1:400, Roche Diagnostics, Mannheim, Germany) (50 μl), secondary antibody, goat anti-rabbit IgG conjugated with peroxidase (diluted 1:1000; Roche Diagnostics, Mannheim, Germany) (50 μl) and the substrate (o-phenylenediamine, Sigma-Aldrich, St. Louis, MO, USA) for color development. Wells were washed with PBS-Tween 20 between incubations. Absorbance at 490 nm was read with a microplate reader (Molecular Devices Corporation, Sunnyvale, CA, USA). Assays were performed in duplicate. The standard curve was prepared with serial dilutions of reference α^1^-AT (human placenta; Sigma-Aldrich, St. Louis, MO, USA). Data were expressed as mole per unit gram of protein.

### Western blot analysis of rat BALF

BALF of rats were subjected to sodium dodecyl sulfate polyacrylamide gel electrophoresis (SDS-PAGE) in a 10% gel under non-reducing condition and electro-blotted onto polyvinylidene fluoride membrane. Non-specific binding was blocked with 5% nonfat dry milk (1 h, 24°C) before the blot was probed for the epitope of interest. Primary antibodies used were those against α_1_-AT (diluted 1:200, rabbit anti-human α_1_-AT; Sigma-Aldrich, St. Louis, MO, USA), NE (diluted 1:200, mouse anti-human NE, Dako, CA, USA), syndecan-1 (diluted 1:200, goat anti-mouse syndecan, Serotec, Raleigh, NC, USA) and secondary antibodies were conjugated with horseradish peroxidase. The membranes were washed with PBS-Tween 20 between incubations. Visualisation was enhanced with a chemiluminescence kit (ECL; Amersham Biosciences, Piscataway, NJ, USA). Bound antibodies were stripped from the membrane by incubation (30 min, 50°C) with 100 mM 2-mercaptoethanol, 2% sodium dodecyl sulfate, 62.5 mM Tris-HCl, pH 6.7, before re-probing with another primary antibody.

### Co-immunoprecipitation

BALF samples (total protein, 300 μg per sample) of COPD patients were pre-cleared with use of protein A-agarose beads (Invitrogen). The supernatant was incubated (16 h, 4 °C) with rabbit anti-syndecan-1 (1 μg; Invitrogen) (test) or rabbit anti-YFP (BD Biosciences (control), on a rota-mixer. A 50% slurry (30 μl) of proteinA-agarose beads was incubated (3 h, 4 °C) to recover the immunoprecipitate in the mixture. After 3 washes with Tris-buffered saline containing 0.1% Tween-20, the beads were heated in reducing sample buffer (10 min, 100 °C) to elute the samples in preparation for SDS-PAGE and Western blotting.

### Casein zymography

Casein zymography of BALF was performed in a 10% PAGE gel containing 1 mg/ml casein under non-reducing condition at 4 °C. After electrophoresis, the gel was washed twice (15 min each) in 2.5% Triton X-100 to remove the SDS. The gel was washed with distilled water several times and then incubated in zymography developing buffer (50 mM Tris–HCl, 200 mM NaCl, 0.02% NaN_3_, pH 7.6) at 37°C for 18 hours. The gel was stained for protein with Coomassie Blue R-250 (0.5% in 40% methanol and 10% acetic acid) and then de-stained in the solvent. Non-staining regions of the gel indicated caseinolytic activity.

### Total and differential cell counts in rat BALF

Bronchoalveolar lavage (BAL) was performed with 1 ml of sterile phosphate-buffered saline. The collected BAL fluid was centrifuged at 200 *g* for 10 minutes at 4°C. The supernatants were stored at −80 °C and the cell pellets were resuspended/diluted to a concentration of 2 × 10^5^ cells/ml. The cell suspension (0.2 ml) was then spun at 1000 rpm for 5 minutes onto a poly-L-lysine-coated glass slide with use of a Cytospin 2 cytocentrifuge (Shandon Instruments, Sewickley, PA, USA). The slides were air-dried, fixed and then stained with Trypan Blue or May–Giemsa stain. Total and differential cell counts of the cytocentrifuge preparations were performed under the microscope with the aid of an eyepiece graticule.

### Immunohistochemical analysis of rat lung section

The largest lobe of the left lung from each animal was excised and then fixed in 4% (w/v) paraformaldehyde, dehydrated with ethanol and embedded in paraffin. The resulting tissue blocks were microtome-sectioned (Leica) to a thickness of 5 μm and then mounted on SuperFrost® glass slides (Menzel-Glaeser, Braunschweig, Germany). The slides were de-waxed, rehydrated and then stained with primary antibody against NE (diluted 1:200, goat anti-NE, Calbiochem, Gibbstown, NJ, USA) or CINC-1 (diluted 1:200, goat anti-CINC, Abcam, Cambridge, MA, USA). This was followed by incubation with HRP-conjugated rabbit anti-goat IgG (diluted 1:100 Serotec, Raleigh, NC, USA). Visualization of the immunohistochemical signals was enhanced with a Dako EnVision + System- HRP (DAB) kit (Dako, Carpinteria, CA, USA). The preparations were counterstained with haematoxylin (Vector Laboratories, Burlingame, CA, USA) and then permanently mounted (BioMeda, Foster City, CA, USA). Images were captured with use of the Olympic IX70 microscope system.

For double immunofluorescence analysis, the slides were de-waxed, rehydrated and stained with primary antibody against syndecan-1 (diluted 1:200, rabbit anti-syndecan-1, Invitrogen, Carlsbad, CA, USA) and CINC-1 (diluted 1:200, goat anti-CINC, Abcam, Cambridge, MA, USA). This was followed by incubation with Donkey anti goat Alex Flour 594 (diluted 1:500, Invitrogen, Carlsbad, CA, USA) and goat anti-rabbit Alex Flour 488 (diluted 1:500, Invitrogen, Carlsbad, CA, USA). Images were viewed and captured with use of the Olympic IX70 microscope system.

### ELISA for cytokines and chemokines

TNF-α, IL-1β and CINC-1 protein concentrations in rat lung homogenates were measured using the rat DuoSet® ELISA Development kits (R&D Systems, MN, USA). The lower detection limits for TNF-α, IL-1β and CINC-1 were 62.5, 31.25 and 4.7 pg/ml respectively.

### Chemotaxis Assay

Both human and rat neutrophils were similarly assessed for chemotactic response to CINC-1 in BALF collected from cigarette smoke exposed rats and sham air controls, a 48-well modified Boyden chamber (Neuroprobe, Gaithersburg, MD, USA) was used as described previously^40^. A total of 2 × 10^5^ neutrophils (from peripheral blood of healthy controls) in a volume of 50 μl were added in the top wells of the chamber. The bottom wells contained 30 μl of the samples to be assayed for chemotactic activity. Samples included recombinant rat CINC-1 (R&D Systems, Minneapolis, MN, USA) in RPMI 1640 medium (Invitrogen, Carlsbad, CA, USA), BALF from smoke-exposed rats and BALF from sham air controls. After incubation of the setup in a CO_2_ incubator (with humidified air with 5% CO_2,_ 37 °C) for 1 hour, the setup was disassembled to recover the filters. The filters were rinsed with PBS to remove surface-lying non-migrating neutrophils. Neutrophils that remained attached to the underside of the filters were fixed with methanol and then stained with Diff-Quick (Fisher Scientific, Pittsburgh, PA, USA). The filters were then mounted onto a glass slide and stained neutrophils were counted under the Olympus IX70 microscope with the aid of an eyepiece graticule. The chemotactic index was expressed as the number of neutrophils that migrated toward a test sample relative to the number of neutrophils that migrated towards a medium control.

### Statistical analysis

Data were expressed as mean ± SD. The INSTAT and PRISM statistical software packages (GraphPad, Inc., San Diego, CA, USA) were used. Data were analysed by one-way analysis of variance (ANOVA) with a Bonferroni’s *post hoc* test or Mann-Whitney U test. Differences are considered statistically significant when P < 0.05.

## Additional Information

**How to cite this article**: Lam, D. C.L. *et al.* *S*-maltoheptaose targets syndecan-bound effectors to reduce smoking-related neutrophilic inflammation. *Sci. Rep.* **5**, 12945; doi: 10.1038/srep12945 (2015).

## Figures and Tables

**Table 1:** Characteristics of subjects recruited.

	Non-COPD Controls	COPD patients	P values
Number of subjects	14	28	
Male Gender	14	28	
Age (years)	62.4 ± 9.3	73.1 ± 5.5	P = 0.065
Smoking status			
Current smokers	6	20	
Ex-smokers	2	8	
Non-smokers	6	0	
FEV_1_% predicted	88.6 ± 10.0	62.4 ± 15.6	P < 0.01
FEV_1_/FVC	75.5 ± 3.7	52.5 ± 13.4	P < 0.01

Data are presented as mean ± SD. COPD: chronic obstructive pulmonary disease; FEV_1_: forced expiratory volume in one second; FVC: forced vital capacity.

**Table 2:** Leucocyte counts and total protein concentration in BALF samples of smoke-exposed rats and sham air controls.

	sham air + neat chitosan	sham air + *S*-maltohepatose-chitosan	cigarette smoke + neat chitosan	cigarette smoke + *S*-maltohepatose-chitosan
Total cells (10^5^/ml)	2.55 ± 0.13	2.80 ± 0.32	9.33 ± 0.43	6.23 ± 0.33
Neutrophils (%)	7.73 ± 0.22	6.98 ± 0.71	24.58 ± 0.75	13.53 ± 1.05
Eosinophils (%)	2.55 ± 0.13	2.90 ± 0.43	0.75 ± 0.13	1.50 ± 0.36
Lymphocytes (%)	24.53 ± 2.86	24.01 ± 4.35	3.73 ± 1.44	18.10 ± 3.34
Macrophages (%)	65.18 ± 2.73	66.11 ± 4.49	70.94 ± 0.70	60.64 ± 3.14
Protein (mg/ml)	0.14 ± 0.04	0.14 ± 0.02	1.08 ± 0.61	0.74 ± 0.05
